# Band Bending Mechanism in CdO/Arsenene Heterostructure: A Potential Direct Z-scheme Photocatalyst

**DOI:** 10.3389/fchem.2021.788813

**Published:** 2021-11-19

**Authors:** Kai Ren, Ruxin Zheng, Jin Yu, Qingyun Sun, Jianping Li

**Affiliations:** ^1^ School of Mechanical and Electronic Engineering, Nanjing Forestry University, Nanjing, China; ^2^ School of Materials Science and Engineering, Southeast University, Nanjing, China; ^3^ School of Automotive and Transportation Engineering, Shenzhen Polytechnic, Shenzhen, China

**Keywords:** two-dimensional, heterostructure, first-principles calculation, Z-scheme, photocatalyst

## Abstract

For the few years, two-dimensional (2D) materials have aroused general focus. In order to expand the properties and application range of 2D materials, two different layered materials are usually combined into heterostructure through van der Waals (vdW) interaction. In this research, based on first-principles simulation, we propose CdO/Arsenene (CdO/As) vdW heterostructure as a semiconductor possessing a direct bandgap by 2.179 eV. Besides, the CdO/As vdW heterostructure presents type-II band alignment, which can be used as a remarkable photocatalyst. Importantly, the CdO/As heterostructure demonstrates a direct Z-type principle photocatalyst by exploring the band bending mechanism in the heterostructure. Furthermore, we calculated the light absorption characteristics of CdO/As vdW heterostructure by optical absorption spectrum and conversion efficiency of a novel solar-to-hydrogen efficiency (*η*_STH_) about 11.67%, which is much higher than that of other 2D photocatalysts. Our work can provide a theoretical guidance for the designing of Z-scheme photocatalyst.

## Introduction

Since graphene was discovered in 2004 (Geim and Novoselov, 2007), it has continuously promoted the research and development of two-dimensional (2D) materials (Miró et al., 2014; Zhong et al., 2019a; He et al., 2019; Sun et al., 2019; Qi et al., 2020; Cui et al., 2021; Dai et al., 2021). After a long time of study on 2D materials, it was found that 2D material has extensive applications and is considered to be one of the most attractive and interesting material fields. All 2D materials show outstanding properties (Vahedi Fakhrabad et al., 2015; Xu et al., 2016; Zhong et al., 2017; Yuan et al., 2018; Sun and Schwingenschlögl, 2020; Luo et al., 2021), for example, the transition metal dichalcogenides (TMDs) materials have remarkable mechanical (Liu and Li, 2015), electronic (Zhang and Singh, 2009), optical (He et al., 2014), magnetic (Yuan et al., 2020) and thermal stability (Ding et al., 2016). Phosphorous possesses novel physical, chemical, optical properties and electrical conductivity (Li and Chen, 2014; Lee et al., 2016). Metal carbide (MXene) has excellent magnetic, thermoelectric properties and carrier mobility. In particular, Cr_2_TiC_2_ monolayer is a new 2D bipolar antiferromagnetic semiconductor and can be used as antiferromagnetic spin field effect transistor (He et al., 2018). The Hf_2_CO_2_ shows the excellent thermal conductivity (about 86.25–131.2 W m^−1^·K^−1^) along the armchair direction, and the expansion coefficient at room temperature is about 6.094 × 10^−6^ K^−1^ (Ren et al., 2021), and the carrier mobility reaches about 1,531.48 cm^2^/V·s (Cai et al., 2014). All these excellent performances explain that 2D materials show potential usage in photocatalysis, photovoltaic devices and heterostructure (Xu et al., 2015; Zhong et al., 2019b; Wang et al., 2020a; Sun et al., 2020b; Wang et al., 2020b; Sun and Schwingenschlögl, 2021a; Sun and Schwingenschlögl, 2021b; Lou et al., 2021; Sun et al., 2021; Zhu et al., 2021).

When TiO_2_ was found to be able to produce hydrogen (H_2_) from ultraviolet irradiated water in 1972 (Fujishima and Honda, 1972), many studies have been carried out using semiconductors as photocatalysts to decompose water (Yuan et al., 2016; Yang et al., 2017; Liu et al., 2018; Wang et al., 2020c; Yong et al., 2020). When the semiconductor is illuminated, the electrons are inspired to move from the valence band maximum (VBM) to the conduction band minimum (CBM), generating holes at the VBM (Maeda and Domen, 2007). However, recompositing rate of photogenerated electron–hole pairs is extraordinary increased due to the simultaneous reduction and oxidation reactions on the surface of monolayer material during water splitting. The popular way to solve this problem is to construct the type-II heterostructure (Ren et al., 2020a), which can effectively separate photogenerated electrons and holes. All 2D heterostructures are formed by van der Waals force (vdW) interaction, which produces more novel properties on the basis of original properties (Ren et al., 2019a), inducing more fantastic optical (Wang et al., 2018), interface properties (Ren et al., 2020b), carrier mobility (Luo et al., 2019) and Gibbs free energy (Ren et al., 2019b). In particular, the Z-scheme photocatalyst has become more and more popular because its special and efficient catalytic mechanism (Xu et al., 2018), such as As/PtS_2_ (Ren et al., 2020c), MoSe_2_/HfS_2_ (Wang et al., 2019), TiO_2_/CdS (Meng et al., 2017) etc., which are proved to possess novel catalytic performance by theoretical and experimental methods. Recently, it has been reported that a hexagonal monolayer semiconductor CdO was prepared by chemical spray pyrolysis and has got a lot of attention due to its outstanding mechanical and stability properties (Subramanyam et al., 1998; Zhuang and Hennig, 2013; Chaurasiya and Dixit, 2019; Chaurasiya et al., 2019; Ali et al., 2021). In addition, heterostructures based on CdO monolayer [such as ZnO/CdO (Sang et al., 2012), CdO/GaS (Zhao et al., 2021), etc.] also demonstrate unusual structural and electronic properties (Sang et al., 2012; Zhao et al., 2021). At the same time, Arsenene (As) is also a 2D material with many special properties, in particular, the band gap can be adjusted by applying external strain on the surface (Kamal and Ezawa, 2015). However, the heterostructures constructed by CdO and As are rarely reported, who share the same honeycomb hexagonal structure. Besides, considering that both CdO and As possess excellent electronic and optical characteristics, it is worth to explore the potential applications of heterostructure based on CdO and As monolayers.

In this study, performing first-principles calculations, the electronic characteristic of the CdO, As and CdO/As heterostructure are investigated with semiconductor nature. Furthermore, the CdO/As heterostructure has a type-II band structure to separate the photogenerated electrons and holes continuously. Interestingly, the bend bending style in CdO/As heterostructure demonstrates a potential direct Z-type photocatalyst and the optical performance is also addressed.

## Materials and Methods

Considering the density functional theory (DFT), all simulation studies in this work were implemented by Vienna *ab initio* simulation software package (VASP) (Capelle, 2006; Togo et al., 2008; Togo and Tanaka, 2015). The core electron is described by projection enhanced wave potential (PAW) (Kresse and Joubert, 1999). The commutative relevant functional was explored, which is introduced by generalized gradient approximation (GGA) and Perdew–Burke–Ernzerhof (PBE) functional (Perdew et al., 1996; Grimme, 2006). At the same time, the weak dispersion force was considered by DFT-D3 with Grimme method (Grimme et al., 2010). Heyd–Scuseria–Ernzerhof mixed functional was used to obtain more accurate electronic and optical properties (Heyd et al., 2003). The parameters of 550 eV and 17 × 17 × 1 were used for the energy cut-off and the Monkhorst–Pack *k*-point grids in the first Brillouin zone. A vacuum space of 25 Å was used in the calculation to keep away from the interaction between adjacent mirror layers. The relaxation of the structure is simulated by conjugate gradient method. The Hellmann–Feynman force on each atom is limited to 0.01 eV Å^−1^.

According to the calculation method of solar-to-hydrogen efficiency (*η*_STH_) proposed by Yang etc (Xu et al., 2016) (*η*_STH_), where *η*_STH_ = *η*_abs_ × *η*_cu_, and *η*_abs_, *η*_cu_ represents light absorption and carrier efficiency, respectively. Besides, the *η*_abs_ is calculated by:

$$\eta_{abs}=\frac{\int_{E_{g}}^{\infty }P\left(h\omega \right)d\left(h\omega \right)}{\int_{0}^{\infty }P\left(h\omega \right)d\left(h\omega \right)}$$ (1)

where *P*(*hω*) is the solar energy flux by AM1.5G with the photon energy *hω*. *E*g is the bandgap of studied materials. Furthermore, the *η*_cu_ is decided by:

$$\eta_{cu}=\frac{\Delta G\int_{E}^{\infty }\frac{P\left(h\omega \right)}{h\omega }d\left(h\omega \right)}{\int_{E_{g}}^{\infty }P\left(h\omega \right)d\left(h\omega \right)}$$ (2)

where Δ*G* is 1.23 eV for the potential difference in water splitting. *E* is the photon energy using for water splitting, which is calculated by:

$$E=\{\begin{array}{c} E_{g},\left(\chi \left(H_{2}\right)\geq 0.2,\chi \left(O_{2}\right)\geq 0.6\right)\\ E_{g}+0.2-\chi \left(H_{2}\right),\left(\chi \left(H_{2}\right)<0.2,\chi \left(O_{2}\right)\geq 0.6\right)\\ E_{g}+0.6-\chi \left(O_{2}\right),\left(\chi \left(H_{2}\right)\geq 0.2,\chi \left(O_{2}\right)<0.6\right)\\ E_{g}+0.8-\chi \left(H_{2}\right)-\chi \left(O_{2}\right),\left(\chi \left(H_{2}\right)<0.2,\chi \left(O_{2}\right)<0.6\right) \end{array}$$ (3)

where *χ*(H_2_) and *χ*( O _2_) are demonstrating the over potential for HER and OER, respectively.

## Results and Discussion

**FIGURE 1:**
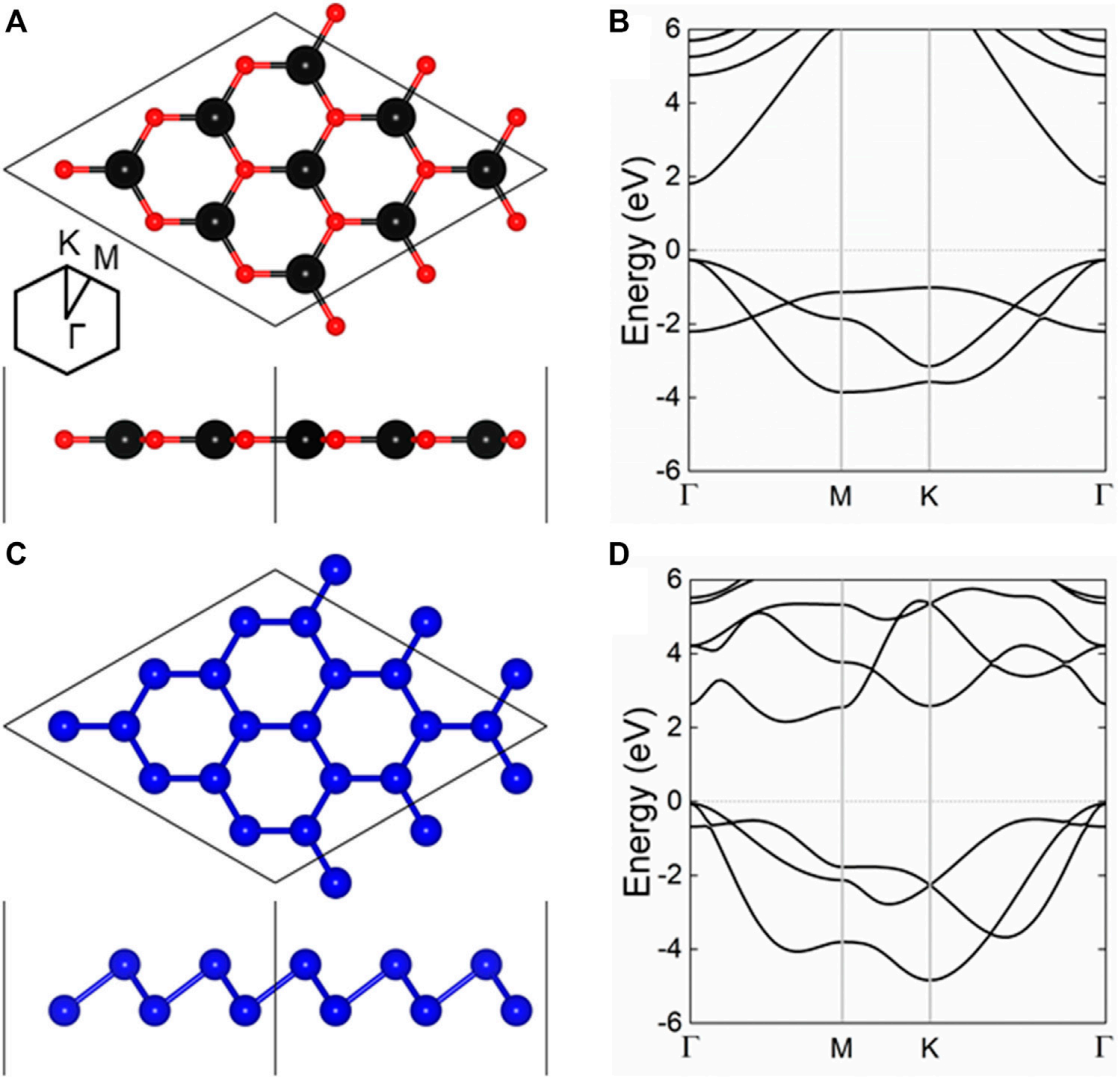
The **(A,C)** crystal structure and the **(B,D)** band structure of the **(A,B)** CdO and **(C,D)** As monolayers; the black, red and blue balls represent Cd, O and As atoms, respectively; the Fermi level is 0 shown as gray dashed line.

First, the crystal structures of single-layer CdO and As was constructed and optimized. The side and top views of CdO and As monolayers are shown in Figures 1A,C, respectively. The lattice constants of CdO and As are calculated to be 3.684 and 3.607 Å, showing a small lattice mismatch of 2.11% for the CdO/As heterostructure, respectively. Besides, the energy band structures of monolayered CdO and As are calculated by HSE06 method, shown in Figures 1B,D, respectively. It can be clearly seen that monolayered CdO and As are semiconductors with the band gaps of 2.073 and 2.234 eV, respectively. For single-layer CdO, the CBM and VBM are located at *Γ* point, showing a direct bandgap structure. While the CBM of As monolayer is located between *Γ* and M points, the VBM exists at Γ points. Besides, the bond lengths of Cd–O and As–As in single-layer CdO and single-layer As were calculated to be 2.127 and 2.506 Å, respectively. Furthermore, all the above calculated results of CdO and As are almost consistent with previous investigations (Ren et al., 2020c; Zhao et al., 2021).

**FIGURE 2:**
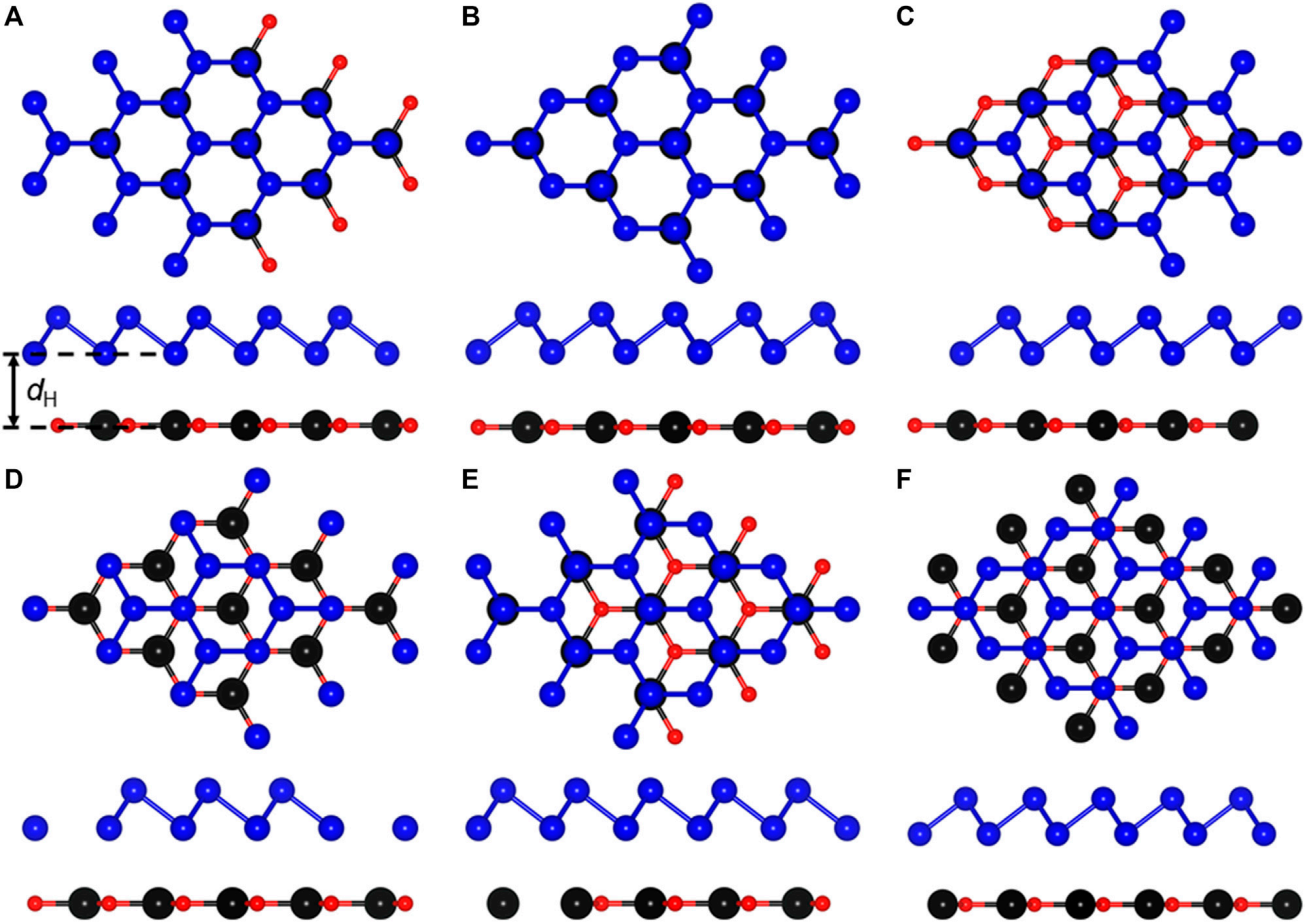
The CdO/As heterostructure constructing by **(A)** CA_1_, **(B)** CA_2_, **(C)** CA_3_, **(D)** CA_4_, **(E)** CA_5_ and **(F)** CA_6_ configurations.

When monolayered CdO and As combine to form a heterostructure, 6 most representative highly symmetrical configurations have be considered. The side and top views of these 6 stacking combinations are shown in Figure 2. Among these 6 heterostructures, the most stable structure is determined by the binding energy (*E*_binding_) between single-layer CdO and As. The investigation shows that the smaller the binding energy is, the more stable the heterostructure is (Singh et al., 2015). The binding energy of CdO/As heterostructures is determined as following:

$$E_{binding}=E_{CdO/As}-E_{CdO}-E_{As},$$ (4)

where *E*_CdO/As_, *E*_CdO_ and *E*_As_ show the total energy of CdO/As heterostructure, single-layer CdO and As respectively. The binding energy of the most stable structure among the 6 stacked heterostructures is −36.64 meV/Å^2^ for the CA_5_ configuration, which is smaller than that in the vdW bonding in weak interlayer interactions in graphites of about −18 meV/Å^2^, shown as Figure 2E, suggesting that there is also a weak vdW force between CdO and As monolayers (Chen et al., 2013). The optimized bond length of Cd−O and As−As in CdO/As heterostructure are 2.082 and 2.504 Å, respectively, which just changed a little comparing with that in CdO and As monolayers, further showing the vdW interaction in CdO/As heterostructure. At the same time, we calculated the different interface distance (*d*_H_) of CdO/As vdW heterostructure, shown in Table 1. Furthermore, the discussed properties of the CdO/As vdW heterostructure is based on CA_5_ stacking configuration.

**TABLE 1:** The binding energy (*E*_binding_, meV/Å^2^), interface distance (*d*_H_, Å) and the bond length (*L*, Å) of the different stacking style CdO/As heterostructure.

	*E*_binding_	*d*_H_	*L*_As–As_	*L*_Cd–O_
CA_1_	−32.07	3.158	2.503	2.082
CA_2_	−28.62	3.334	2.509	2.083
CA_3_	−32.67	3.119	2.501	2.082
CA_4_	−28.19	3.332	2.508	2.084
CA_5_	−36.64	2.892	2.504	2.082
CA_6_	−35.17	2.972	2.505	2.083

**FIGURE 3:**
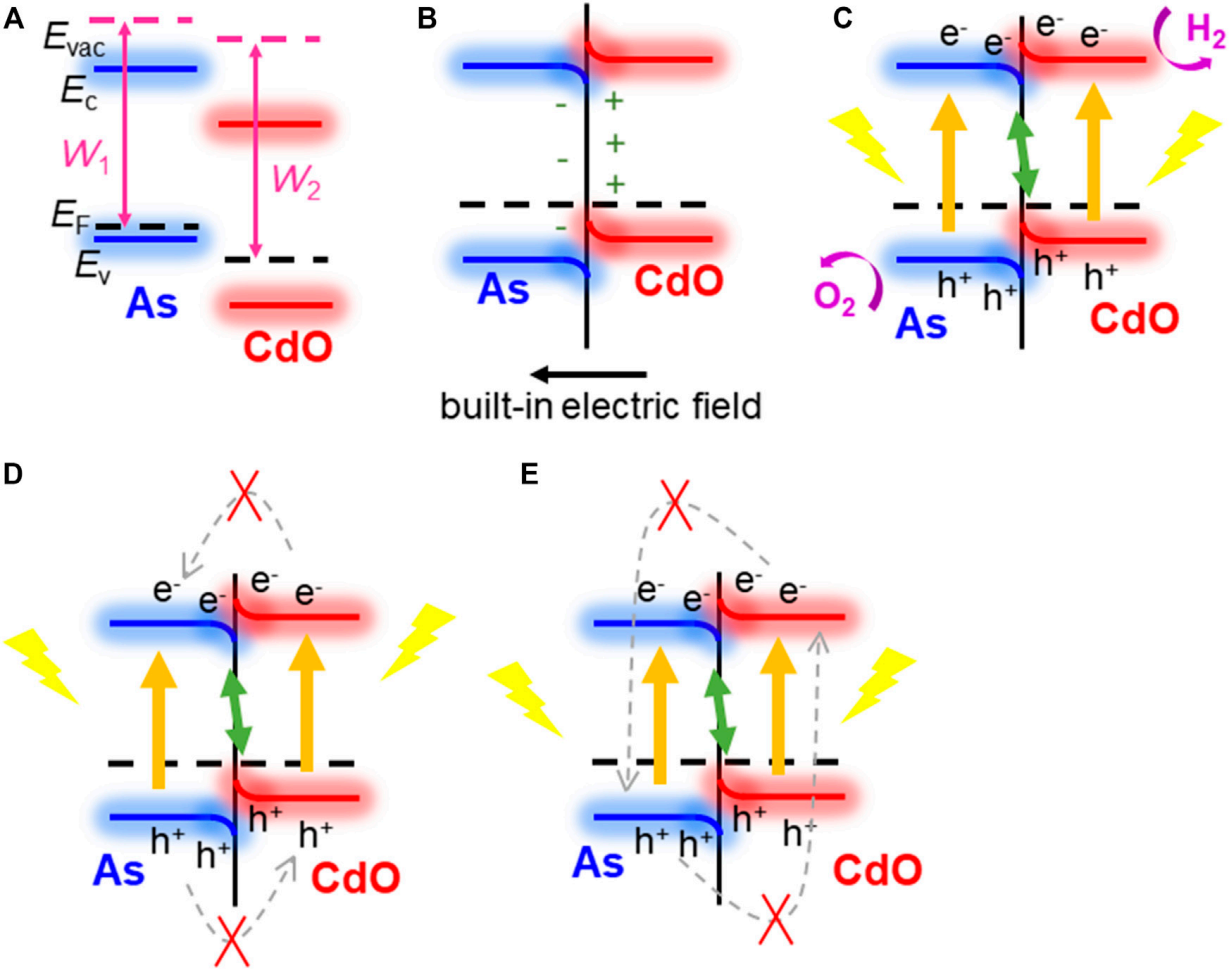
**(A)** The projected band structure and **(B)** the band-resolved charge densities of the CdO/As vdW heterostructure; the Fermi level is zero energy indicated by gray dashed line. **(C)** Schematic of the migration for the CdO/As vdW heterostructure using as a photocatalyst.

The projected band structure of CdO/As vdW heterostructure is calculated using HSE06 method, shown Figure 3A. Obviously, it can be clearly seen that CdO/As vdW heterostructure demonstrates the nature of semiconductor and shows a direct bandgap of 2.179 eV. Besides, it also can be seen that the CBM and VBM of CdO/As vdW heterostructure are located as *Γ* point contributed by As and CdO monolayers, respectively, which reveals a type-II band style. Then, such type-II band structure is further proved using the band-resolved charge densities for the CdO/As vdW heterostructure shown in Figure 3B. When the CdO/As vdW heterostructure is illuminated by the light, expressed by Figure 3C the photogenerated electrons will move from the VB of both CdO and As monolayers to the CB and the holes are keep. Then, by the assistance of the valence band offset (conduction band offset), the photogenerated electrons (holes) at CB (VB) of the CdO (As) layer migrate to the CB (VB) of the As (CdO) layer, thus, the photogenerated electrons and holes are effectively separated. Therefore, the gained type-II band alignment of CdO/As vdW heterostructure can effectively resist the recomposite of photogenerated electrons and holes, showing potential candidate use in application as a photocatalyst for water splitting.

**FIGURE 4:**
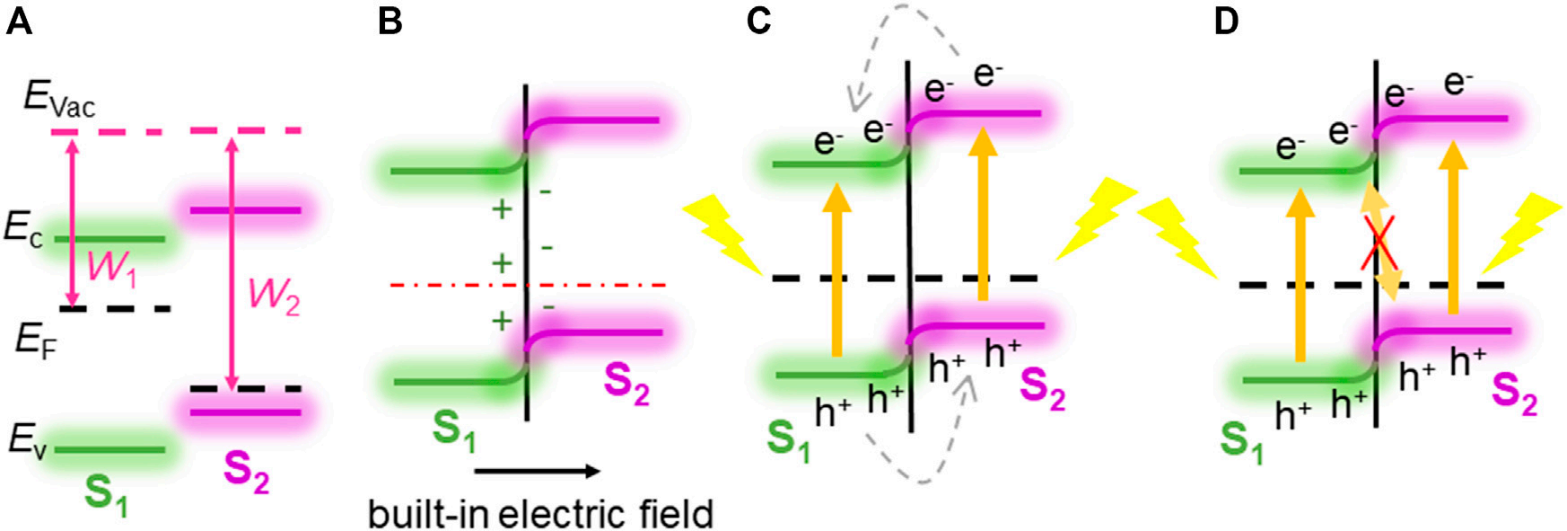
The direct Z-scheme mechanism demonstration for CdO/As vdW heterostructure: **(A)** before combining, **(B)** in combining; **(C–E)** photoinduced charge carrier migration process.

Next, we explain how the direct Z-scheme structure can be used as a photocatalyst in CdO/As vdW heterostructure. It is of great significance to calculate the work function (*W*) difference between single-layer CdO and single-layer As, which is a prerequisite for driving charge redistribution and forming built-in electric field through CdO/As vdW heterostructure interface (Bai et al., 2015; Liu et al., 2016). Shown in Figure 4A, Before the intercourse of single-layer CdO and single-layer As, the work functions of CdO (*W*_2_) and As (*W*_1_) are calculated to be 5.783 and 5.443 eV respectively. It can be seen from the calculation results that *W*_1_ is less than *W*_2_. According to the electron transfer mechanism, it can be concluded that electrons will be transferred from CdO layer to As layer until the Fermi level conforms to the equilibrium of Anderson rule (Zhang and Yates, 2012), shown in Figure 4B. Due to the transfer of electrons from CdO layer to As layer, positive holes are left in CdO layer, while negative electrons are accumulated in As layer, and a built-in electric field is generated at the interface. Subsequently, the electrons in the CdO layer and the negative charges in the As layer repel each other, which leads to the upward bending of the CdO band and the downward bending of the As layer at the interface for the same reason (Zhang and Yates, 2012; Huang et al., 2017). After photon excitation, both CdO and As can induce electrons and holes, as shown in Figure 4C. In this case of band bending, it is best to use the direct Z-scheme to transform the structure (Xu et al., 2018). The bending mode and built-in electric field of the band support the recomposite of light photogenerated holes in the VB of the CdO and photogenerated electrons in the CB of the As. Furthermore, this built-in electric field and extra potential barrier, which is also generated by band bending, will obstacle the flowing of the photogenerated electrons from CB of the CdO to the As, and the photogenerated holes from VB of the As to CdO, shown as Figure 4D. The built-in electric field also has ability to prevent the recomposite of the photogenerated electron in the CB of the CdO to the holes in the VB of the As, explained as Figure 4E. Therefore, the CdO/As vdW heterostructure can be considered as a potential direct Z-type photocatalyst in water splitting.

**FIGURE 5:**
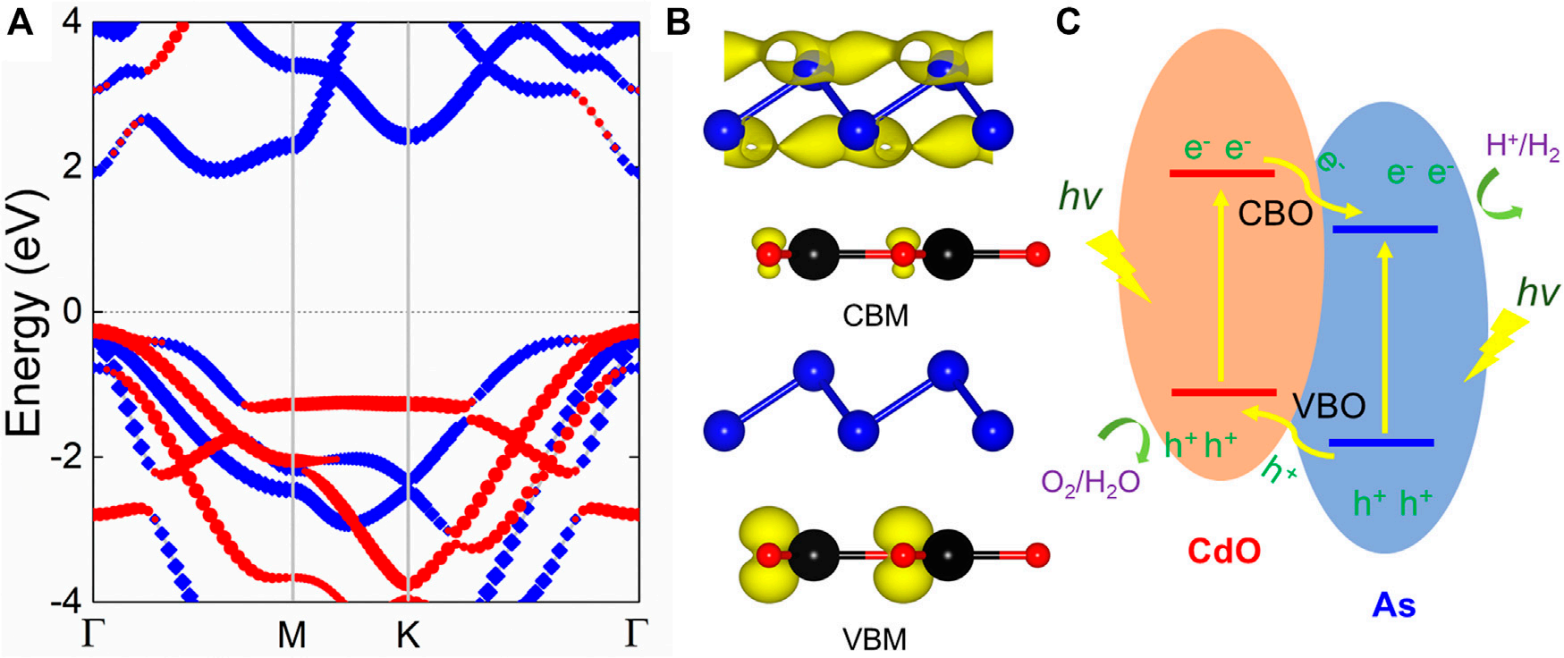
The p–n heterostructure schematic illustration: **(A)** before combining, **(B)** in combining, **(C)** transformation of photogenerated charge carriers in p–n heterostructure, and **(D)** suppressed transformation of photogenerated charge carrier in direct Z-scheme style.

However, the process that the built-in electric field generated by the band bending trend inducing the photogenerated electrons and holes moving mode provides the Z-scheme photocatalytic mechanism for CdO/As vdW heterostructure to decompose the water is not coincidental. It is contributed form the critical band bending trend of the CdO/As vdW heterostructure. In contrast, another band bending method, such as p–n heterostructure, will not result the Z-scheme photocatalytic path for the photoinduced charges. As shown in Figure 5A, when the heterostructure is formed by n-type (work function of *W*_1_) and p-type semiconductors (work function of *W*_2_), the *W*_1_ is smaller than *W*_2_, free electrons can move from n-type material to p-type material, inducing the band of the n-type semiconductor bending upward, while the band of the p-type semiconductor bending downward across the interface of the heterostructure. Subsequently, the built-in electric field is constructed, as shown in Figure 5B. Under this built-in electric field assistances, the electrons at the CB of the p-type material will prefer moving to the CB of the n-type material, and the photogenerated holes at the VB of the n-type semiconductor will choose to migrate to the VB of the p-type semiconductor (Figure 5C). Moreover, even the band alignment of this heterostructure satisfy the band edge positions of the Z-scheme photocatalyst, the built-in electric field resulted by this band bending trend will not boost a combination for the photoinduced electrons at the CB of the n-type semiconductor and the photoinduced holes at the VB of the p-type semiconductor (Figure 5D) (Xu et al., 2018). Therefore, the direct Z-scheme mechanism is an intrinsic property of the CdO/As vdW heterostructure.

**FIGURE 6:**
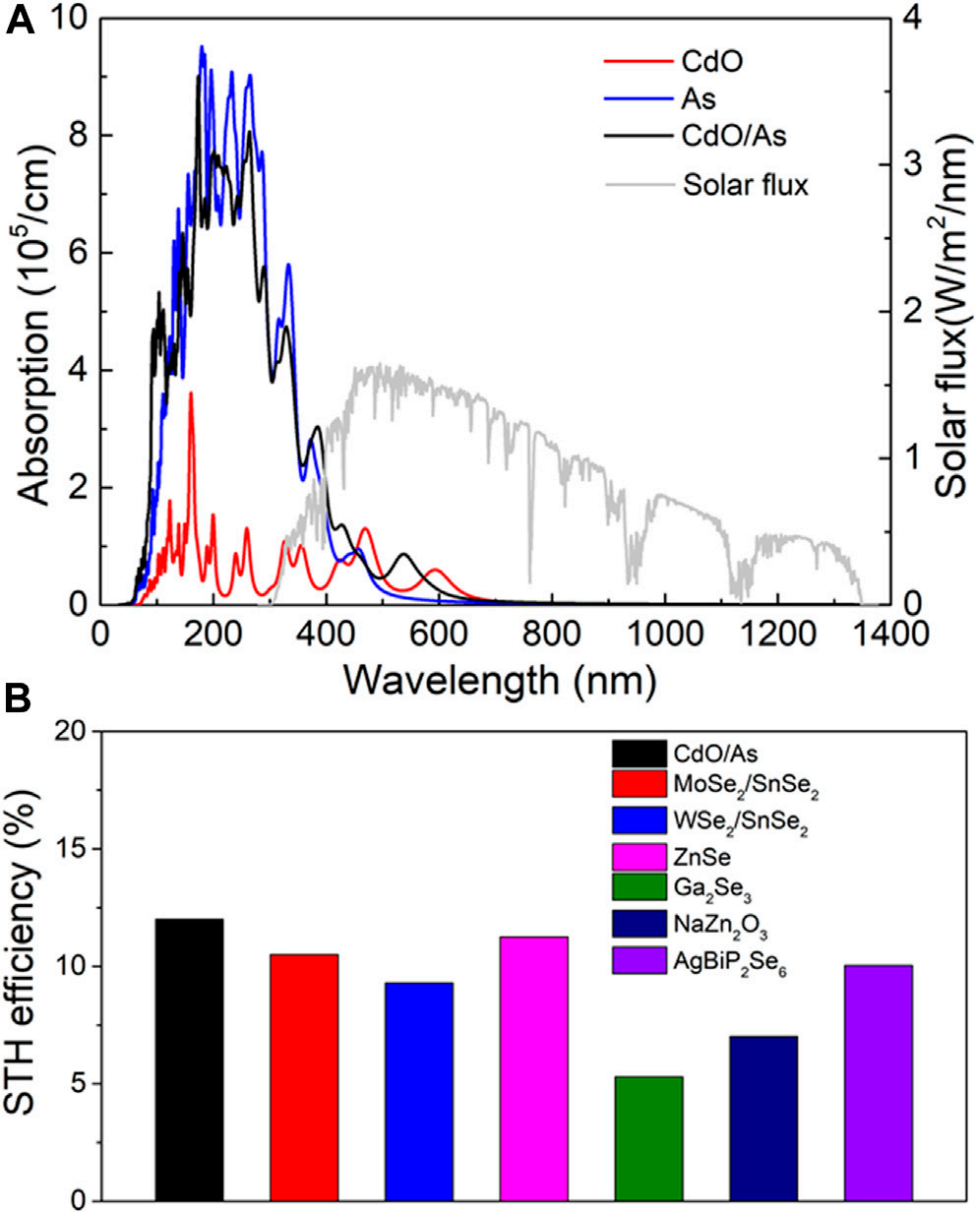
**(A)** The HSE06 method obtained optical absorption spectrum and **(B)** the STH efficiency of CdO/As vdW heterostructure comparing with other 2D materials (Fan et al., 2019; Jin et al., 2019; Yang et al., 2019).

As a potential candidate for direct Z-scheme photocatalyst to decompose water, the optical property is essential performance to be assessed. The optical absorption spectrum of the CdO, As and CdO/As vdW heterostructure are calculated in Figure 6A, which evidently explain the CdO/As vdW heterostructure can improve the visible light absorption capacity (wavelength range 380–800 nm). The obtained excellent absorption peak of the CdO/As vdW heterostructure is 8.47 × 104 cm^−1^ at the wavelength of 542 nm. Besides, enhancing solar energy conversion efficiency is the ultimate target for that, which demonstrates the indeed usage of solar energy for HER and OER (Lu et al., 2019). Therefore, we calculated STH efficiency (*η*_STH_) for the CdO/As vdW heterostructure. The obtained *η*_abs_ and *η*_cu_ are 58.1 and 20.1%, respectively. The *η*_STH_ of the monolayered CdO, As and CdO/As vdW heterostructure is also calculated in the Table 2. The obtained *η*_STH_ of the CdO/As vdW heterostructure as 11.67% indicates such Z-scheme photocatalyst possesses a novel STH efficiency, which is also higher than other reported photocatalysts, shown in Figure 6B. It worth noting that we assumed the 100% efficiency of the catalytic reaction for the calculations of the STH efficiency (Fu et al., 2018).

**TABLE 2:** The energy conversion efficiency of light absorption (*η*_abs_), carrier utilization (*η*_cu_) and STH (*η*_STH_) of the monolayered CdO, As and CdO/As vdW heterostructure.

2D materials	*η*_abs_ (%)	*η*_cu_ (%)	*η*_STH_ (%)
CdO	63.3	22.6	14.3
As	36.4	28.4	10.3
CdO/As	58.1	20.1	11.67

## Conclusions

Based on the first-principles calculation, firstly, we systematically studied the geometry and band structure of single-layer CdO and As. Then, the CdO/As heterostructure is constructed using vdW forces possessing a direct bandgap as 2.179 eV and a type-II band alignment structure is realized, which can limit the recomposite of photogenerated electron−hole pairs. Next, the band bending configuration of CdO/As vdW heterostructure is addressed, which demonstrates the potential Z-scheme conversion mechanism using as a photocatalyst for HER and OER. Furthermore, the excellent *η*_STH_ of CdO/As vdW heterostructure is obtained by 11.67%. All our results show that the CdO/As vdW heterostructure can be used as a potential direct Z-scheme photocatalyst for water splitting.

## Data Availability

The raw data supporting the conclusion of this article will be made available by the authors, without undue reservation.
